# Single Honeybee Silk Protein Mimics Properties of Multi-Protein Silk

**DOI:** 10.1371/journal.pone.0016489

**Published:** 2011-02-02

**Authors:** Tara D. Sutherland, Jeffrey S. Church, Xiao Hu, Mickey G. Huson, David L. Kaplan, Sarah Weisman

**Affiliations:** 1 Entomology Commonwealth Scientific and Research Organisation (CSIRO), Canberra, Australia; 2 Materials Science and Engineering CSIRO, Geelong, Victoria, Australia; 3 Department of Biomedical Engineering, Tufts University, Medford, Massachusetts, United States of America; Massachusetts Institute of Technology, United States of America

## Abstract

Honeybee silk is composed of four fibrous proteins that, unlike other silks, are readily synthesized at full-length and high yield. The four silk genes have been conserved for over 150 million years in all investigated bee, ant and hornet species, implying a distinct functional role for each protein. However, the amino acid composition and molecular architecture of the proteins are similar, suggesting functional redundancy. In this study we compare materials generated from a single honeybee silk protein to materials containing all four recombinant proteins or to natural honeybee silk. We analyse solution conformation by dynamic light scattering and circular dichroism, solid state structure by Fourier Transform Infrared spectroscopy and Raman spectroscopy, and fiber tensile properties by stress-strain analysis. The results demonstrate that fibers artificially generated from a single recombinant silk protein can reproduce the structural and mechanical properties of the natural silk. The importance of the four protein complex found in natural silk may lie in biological silk storage or hierarchical self-assembly. The finding that the functional properties of the mature material can be achieved with a single protein greatly simplifies the route to production for artificial honeybee silk.

## Introduction

Silks are extraordinary materials produced by a great number of spiders and insects for many different functions. The versatility of silk together with its fabrication from aqueous solutions at ambient temperatures and pressures have led researchers to invest considerable efforts into mimicking the material. The silks that have received the greatest attention are the dragline silk of spiders and the cocoon silk of silkworms—the spider silk by virtue of its remarkable tensile strength, the silkworm silk because of its long history as a luxury fabric. However, the ability to produce silk has arisen independently many times [1] and there is increasing focus on exploring the potential of silks from different evolutionary lineages.

**Figure 1:**
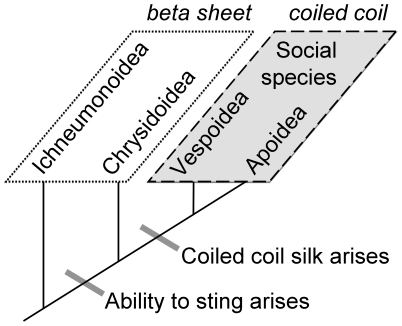
Schematic of the evolutionary relationship of Hymenopteran superfamilies that have evolved sociality, showing the molecular structure of crystallites in the silks they produce. Protein structural information from Rudall and Kenchington (1971) and Sutherland et al. (2006).

Of particular interest is the silk of the social Hymenoptera: the bees, ants and hornets. The principal molecular structure of social Hymenopteran silk is α-helical proteins assembled into a tetrameric coiled coil conformation [2], [3], a fundamentally different design to the β-sheet crystallites that dominate the silkworm cocoon and spider dragline silks. Silks with β-sheet crystallites are plesieomorphic (the ancestral state) in the Hymenoptera. Similarly structured silks continue to be produced by insects from the Ichneumonoidea [2], the sister superfamily to the stinging insects, as well as by stinging insects from the Chrysidoidea [4], the non-social sister superfamily to the social superfamilies Apoidea and Vespoidea (Figure 1). Two key prerequisites to the evolution of sociality are the ability to construct nests to house multiple generations and the ability to protect juveniles within the nest [5]. Sociality in the Hymenoptera has occurred only in lineages that have both evolved the ability to sting and developed coiled coil silk. The coincidence of sociality and coiled coil silk implies that evolution of a coiled coil silk has contributed to the huge evolutionary success of the social Hymenoptera.

The features of coiled coil silk that make it attractive as a nest material are not yet known, although information about the mechanical and functional properties of the native material is accumulating. Measurements of the mechanical properties of honeybee silk fibers hand-drawn from *Apis mellifera adansonii* (African honeybee) larvae show greater toughness and extensibility (204%) but reduced tensile strength (132 MPa) compared to spider dragline and silkworm cocoon silks [6]. More broadly, bee silk has been shown to contribute to the thermal and mechanical stability of *Apis mellifera scutellata* [7] and *Apis mellifera Ligusicua* [8] hives.

The genes encoding the coiled coil silk proteins of three bee species (superfamily Apoidea), two ant and a wasp species (superfamily Vespoidea) have been described [4], [9], [10], [11]. Each species contains four paralogous silk genes with a copy of each paralog identified in all species. The genes encoding each paralog are expressed equivalently in the silk gland [4]. The proteins encoded by the homologs are very similar in amino acid composition and molecular architecture (each containing an extensive coiled coil domain flanked by hydrophilic termini) suggesting functional redundancy. However, Apoidea diverged from Vespoidea over 150 million years ago and if the genes were functionally interchangeable gene duplication or deletion events would be expected in some lineages. The conservation of a copy of the four paralogs in all species over such a long time period implies a unique role for each protein in either silk fabrication or silk function.

Silk fabrication has been investigated in honeybees, bumble bees and colonial wasps [12]. Silk proteins are produced by modified salivary glands (labial glands) and secreted into the gland lumen where they accumulate to high concentrations during the final larval instar. The honeybee proteins assemble into fibrils approximately 4.0–4.5 nm in width, with some evidence that these fibrils are comprised of pairs of fine filaments each 2.0–2.5 nm wide—the expected diameter of the tetrameric coiled coil structures of the silk proteins [3]. The fibrils further assemble into tactoids 1–3 µm in width and 3–40 µm in length [12]. In polarised light, these tactoids are strongly birefringent with the principal axis parallel to the length of the tactoid, indicating that the fibrils are aligned in that direction and oriented in the direction of flow out of the silk gland. Macromolecular organisation was also observed within the labial gland of bumble bees and colonial wasps [12].

In contrast to the large (>200 KDa) and repetitive proteins that dominate the silkworm and spider dragline silks, the coiled coil silk proteins are small (30–40 KDa) and non-repetitive [4], [9], [10], [11]. Full-length tagged [11] and untagged [13] recombinant silk proteins have been produced by fermentation in *Escherichia. coli* and the untagged proteins have been fabricated into materials with coiled coil structure that mimic the native material [13]. Silken threads with comparable extensibility to native silk have been hand-drawn from concentrated solutions of mixtures of all four recombinant silk proteins [13]. Drawing these threads a second time achieved tensile strengths similar to the native silk fibers but with reduced extensibility. Threads could only be fabricated by this technique from solutions containing all four recombinant honeybee silk proteins, with single protein solutions precipitating as the solutions were concentrated.

In the present study the goal was to determine whether all of the four related coiled coil silk proteins, which have been conserved for over 150 million years, are essential to silk material function. A silk production method was developed that allows us to spin fibers consisting of only a single recombinant protein. A range of materials characterization techniques was used to investigate the solution behavior, solid-state structure, and mechanical properties of material containing a single honeybee silk protein (AmelF3) and compare these to the properties of native silk or materials generated from a mixture of all four recombinant proteins (AmelF1-4).

## Results and Discussion

### Single honeybee proteins form coiled coils in solution

The AmelF3 honeybee silk protein was expressed into the inclusion bodies of *E. coli*—a method that allowed rapid and simple recovery of relatively pure proteins from the fermentation mixture [13]. The F3 paralog was chosen for this study because although the other paralogs could be expressed at high levels [13] only AmelF3 remained in solution when concentrated in isolation. AmelF3 inclusion bodies were unfolded using equivalent dry weight of the detergent sodium dodecyl sulfate (SDS) to generate 2–4% monomeric protein solutions. Dynamic light scattering (DLS) measured the hydrodynamic diameter of particles in the protein-detergent solution diluted ten-fold in 100 mM NaCl as 9.2+/−0.1 nm (peak containing 98.2% of particle volume). The diameter of SDS micelles in 3% SDS solutions without protein under the same experimental conditions was a single peak at 5.5+/−0.2 nm. No SDS micelles were detected in the SDS–protein solutions confirming that the majority of the SDS was bound to the protein, as expected from the literature [14].

**Figure 2:**
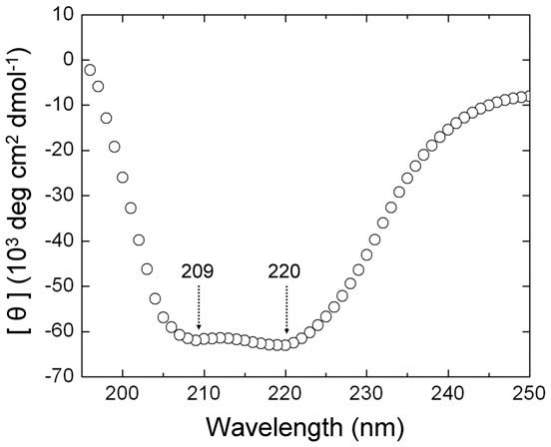
Circular dichroism spectrum of a solution of AmelF3 protein, with minima at 220 and 209 nm indicating the protein is present in a coiled coil structure.

Proteins were refolded by removing SDS using KCl. Potassium dodecyl sulfate has significantly lower solubility than SDS and precipitates out of solution where it can be removed by centrifugation. The protein solutions were dialysed against water to reduce salt levels then concentrated by dialysis against PEG8000, resulting in 3–4% protein, 0.2–0.4% SDS concentration and 15 mM NaCl-KCl at pH 7–7.2. Initial DLS experiments on the protein solutions after removal of detergent detected only large particles (>100 nm) with no reproducibility between measurements. Similarly it has been reported that poor DLS data sets were obtained with homologous proteins from *Apis cerana* when the proteins were concentrated above 0.15% [11]. When AmelF3 solutions were diluted ten-fold in 100 mM NaCl (comparable to physiological salt levels) the particle diameter was measured as 20.3+/−0.7 nm (peak containing 86.8% of particle volume), in reasonable agreement with the approximate particle diameter calculated for an AmelF3 coiled coil (see methods section). The dramatic improvement in data quality is possibly because the salt ions out-competed salt bridges or charged residue interactions that were promoting non-specific association of the proteins. The circular dichroism spectra of AmelF3 solutions showed strong spectral minima at 220 and 209 nm and a 220 nm/209 nm ratio of 1.02 supporting a coiled coil structure (Figure 2). A 220 nm/209 nm ratio of one or more is indicative of coiled coils whereas a ratio of less than 0.86 is indicative of isolated helices [15]. DLS measurements indicated that after addition of SDS back to the AmelF3 solutions the hydrodynamic particle diameter was reduced to the size observed in the original monomeric solutions, confirming that the removal of most SDS is a prerequisite for the protein to fold into a native-like silk protein conformation.

In contrast to the AmelF3 protein, His-tagged recombinant versions of the homologous protein from *Apis cerana* remained monomeric and predominantly random coil at comparable concentrations [11]. Protein purification tags have been shown to have a significant effect on secondary structure of recombinant spider silk proteins [16], possibly explaining the different solution behaviour of the *Apis cerana* homologs. Alternatively, the initial presence of SDS may be assisting coiled coil formation of the honeybee silk proteins. Although it is commonly held that addition of SDS leads to unfolding of proteins in solution, SDS has been shown to promote the formation of secondary structure in many proteins [14], [17], [18]. For example, the component proteins of the leucine zipper GCN4 are stabilised as individual α-helices at SDS concentrations above 1 mM [19]. If the AmelF3 proteins are stabilised as α-helices by the presence of SDS then this may promote coiled coil formation as the SDS is removed.

### A single silk protein is sufficient for native-like structure and mechanical properties

**Figure 3:**
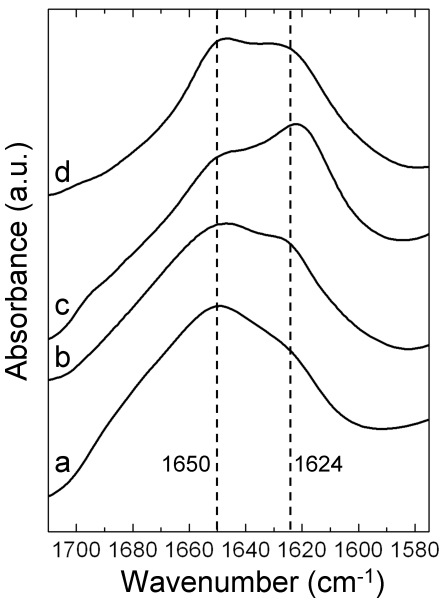
The amide I regions of Fourier transformed infrared (FTIR) spectra from AmelF3 films and native silk. a. AmelF3 film, b. AmelF3 film after heating to above T*g*, c. AmelF3 film after MeOH treatment, d. native honeybee silk.

The molecular structure of native social Hymenoptera silk contains both coiled coils and β-sheets with the proportions differing in silks from various species. For example, amino acid sequence analysis predicts that around 46% of the hornet silk is coiled coil [10] in comparison to around 65% in honeybee silk [9], and IR and NMR measurements show that the β-sheet content of native hornet silk is significantly greater than that of honeybee silk [9], [20], [21]. In order to determine the molecular structure of solid AmelF3 material, solutions of AmelF3 were dried into films and analysed by Fourier transform infrared spectroscopy (FTIR) (Figure 3). The infrared spectrum obtained from these films had maxima in the amide I region at 1650 cm^−1^ and 1624 cm^−1^ (Figure 3). Absorption bands in the frequency range of 1610–1625 cm^−1^ are expected to represent β-sheet structure [22], [23], whereas absorption bands in the frequency range of 1640–1650 cm^−1^ are ascribed to the overlapping peaks at 1652 cm^−1^, 1644 cm^−1^, and 1632 cm^−1^ of the coiled-coil fingerprint spectrum [24]. The features seen in the recombinant AmelF3 film (Figure 3) are consistent with the coiled coil structure found in native honeybee silk and in films generated from all four recombinant honeybee silk proteins [13]. The levels of β-sheet, correlated to features at 1624 cm^−1^, were considerably less than those seen in native hornet silk [21].

Thermal [25] and methanol [26], [27], [28] treatments have been shown to induce β-sheet formation in reprocessed silkworm silk. The responses of AmelF3 protein films to thermal and methanol treatment were investigated by infrared spectroscopy and compared to the behavior of native silk and material generated from all four recombinant proteins. Previously, it was shown that methanol could induce partial β-sheet formation in recombinant honeybee silk films generated from all four silk proteins [13]. Infrared spectroscopy indicated that methanol treatment also induced β-sheet formation in AmelF3 films, with an increase in the feature at 1624 cm^−1^ (Figure 3). Differential scanning calorimetry (DSC) and temperature-modulated differential scanning calorimetry (TMDSC) were used to determine the glass transition temperature (T*g*) of AmelF3 films. TMDSC can eliminate the non-reversing thermal phenomena of the protein sample such as water evaporation or physical aging and clearly reveal the glass transition regions. The analysis of thermograms obtained from both DSC and TMDSC of AmelF3 films yield a T*g* of 184°C. Films heated to above the T*g* and then cooled were analysed by IR spectroscopy. Heating appeared to enforce the self-assembly of molecular structure as AmelF3 films heated to 215°C gave clearer beta-sheet features at 1624 cm^−1^ than those of the untreated films (Figure 3).

**Figure 4:**
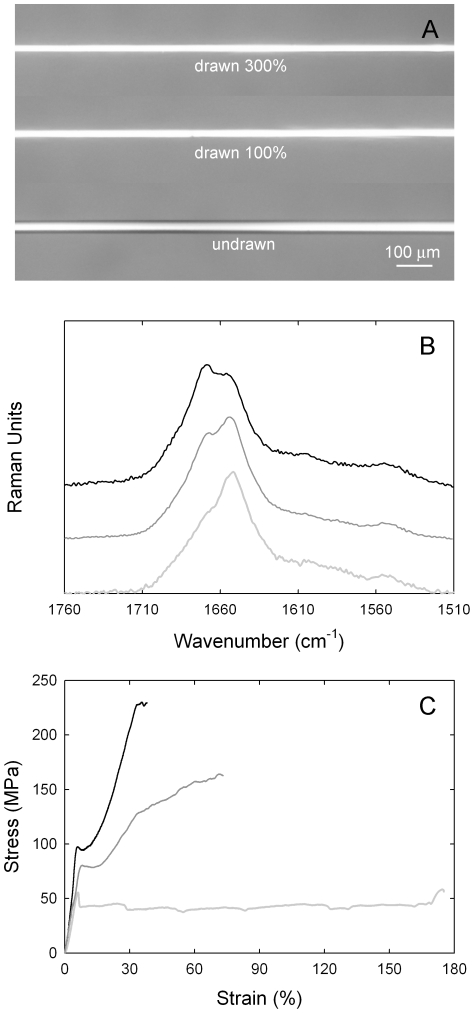
Fibers under cross polarised light (A) Micro Raman spectra (B) and stress-strain plots (C) of representative recombinant honeybee silk fibers (mixture of AmelF3 and AmelF1-4 data). Fibers were either undrawn (pale grey lines) or wet-drawn to around 100% (dark grey lines) or 300% (black lines) their original length in a 90% methanol bath.

Fibers of AmelF3 protein were generated by extruding solutions of approximately 3% AmelF3 and less than 0.4% SDS through capillary tubing into an aqueous methanol coagulation bath. The mechanical properties of the single protein fibers were compared to fibers spun by the same process from equimolar solutions of all four honeybee silk proteins (AmelF1-4). As native fibers are laid down as mats with fibers partially merged, they are not suitable for comparative analysis. Air-dried recombinant fibers were further processed by wet-drawing in an aqueous methanol bath to between two to four times their original lengths (Figure 4A). In the methanol bath the dried threads were translucent and they became opaque during wet-drawing, consistent with the formation of ordered regions and similar to what was observed in fibers drawn from concentrated protein solutions into the air [13]. In contrast to films generated from reconstituted hornet silk [29], the fibers did not become opaque upon wetting but only during the drawing process.

The mechanical properties of AmelF3 fibers and AmelF1-4 fibers at set temperature and humidity were very similar (Table 1). For both fiber types, drawing had the effect of increasing the strength and stiffness and decreasing the extensibility (Table 1). Although the AmelF1-4 fibers generally had slightly higher strength and less extensibility than the AmelF3 fibers for the same nominal draw ratio, the comparison is complicated by the fact that the initial undrawn diameters and the actual draw ratios (Table 1) are slightly different. Fracture mechanics theory predicts an increase in strength with decreasing diameter and increasing the draw ratio also leads to an increase in strength. If we compare strengths at the same diameter then AmelF3 appears to be about 50–60% stronger than AmelF1-4, however the undrawn AmelF3 fibre was coarser and hence it required more drawing to get to the same diameter. If we plot the data against the calculated draw ratio then the difference between the fibres is greatly reduced. On balance, the results suggest that changing the composition of the fibres from a mix of four proteins to a single protein does not significantly alter the mechanical properties.

**Table 1:** Mechanical properties of honeybee silk fibers.

Method of fabrication	Constituent proteins	Diameter (µm)	Draw ratio^1^	Breaking stress (MPa)	Breaking strain (%)	Toughness (MPa)
Extruded into 70% MeOH	AmelF1-4	31±2	0	70±4	190±11	91±7
	AmelF3	45±2	0	50±3	243±10	105±6
Extruded into 70% MeOH then drawn ≈100% in 90% MeOH	AmelF1-4	21±1	2.2	133±11	94±11	85±9
	AmelF3	34±2	1.8	97±7	129±15	97±10
Extruded into 70% MeOH then drawn ≈300% in 90% MeOH	AmelF1-4	17±1	3.3	203±10	51±5	70±8
	AmelF3	23±1	3.8	178±20	68±9	85±18
Drawn in air^2^	AmelF1-4	30±5	NG	15±3	225±10	NG
Drawn in air then in 90% MeOH (13)	AmelF1-4	13±7	NG	150±39	47±26	NG
*Natural* (7)	*Native*	*9*	NG	*132*	*204*	NG

1 Calculated as (d_0_/d_1_).

2 where d_0_ and d_1_ are the diameters of the initial and drawn fibres.

NG: not given.

Micro-Raman analysis confirmed that wet-drawing of the fibers led to changes in the protein conformation with similar trends observed in both sets of fibers. Representative spectra are shown in Figure 4B. The undrawn fibers had amide I band maxima at 1654 cm^−1^ characteristic of α-helix [30] and 1669 cm^−1^ characteristic of β-sheet [30]. Wet-drawing led to the β-sheet feature increasing in intensity. There was no evidence of residual SDS in the fibers by with Raman showing no aliphatic C-H stretching vibrations at 2882 and 2848 cm^−1^ [31] and infrared showing no aliphatic C–H stretching modes at 2918 and 2851 cm^−1^ or sulfate bands near 1237 cm^−1^ [31].

In addition to having essentially equivalent mechanical properties at break (discussed above), at each level of draw the AmelF3 and AmelF1-4 fibers had similarly shaped stress-strain curves. Three distinct regions of extension were observed, most clearly in fibers drawn approximately 100% (Figure 4C). Initially, up to about 7% strain, a linear elastic Hookean region was evident. In semi-crystalline polymers this region is associated with covalent bond stretching and distortion. After the linear region there is a yield region in which the stress suddenly decreases and stretching or drawing of the fibre occurs at constant stress. This stage was accompanied by the formation and extension of a neck in the fibers. In semicrystalline polymeric materials, necking behaviour is associated with rearrangement of the molecules to produce a structure (both amorphous and crystalline) that is oriented in the direction of the stress. In α-keratin materials (wool, hair, quills, horns), which also display three distinct stress-strain regions [32], the yield region is believed to involve the transformation of the α-helices to β-pleated sheet [33], [34], [35]. In the recombinant silk threads this region is most pronounced in undrawn threads, which contain the highest levels of α-helix, and is reduced in fibres drawn approximately 100% and further reduced in fibres drawn 300%. We interpret this region as corresponding to development and alignment of a β-sheet network as shear forces bring the N- and C- termini sections of the proteins into close proximity, as well as increasing alignment of the coiled coil regions. Finally a third, post-yield, stress-strain region was observed where chains become load-bearing and the stress increases up until failure occurs. This region was extensive in drawn fibers rich in β-sheets (Figure 4B, drawn 300%), reduced in fibers containing less β-sheets (Figure 4B, drawn 100%) and almost non existent in the undrawn sample. The virtual absence of a post-yield region in the undrawn sample suggests that cold drawing in air (during the tensile test) is far less efficient in the transformation of the coiled coils to β-sheets than wet drawing in 90% methanol. The change in slope seen in this region for the 100% drawn fibres is curious and may involve the further transformation of residual α-helical material. We are currently undertaking a detailed analysis of the structural transformations occurring in fibers under load.

**Figure 5:**
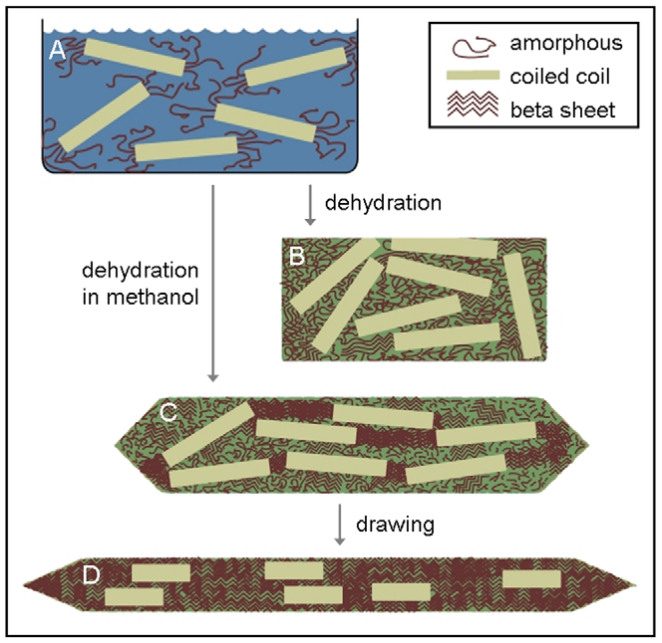
Schematic showing molecular structural changes occurring in recombinant AmelF3 protein during material fabrication. **A**. Silk proteins adopt coiled coils in solution; **B**. When dehydrated in air, the dominant molecular structure remains predominantly coiled coil; **C**. When dehydrated in methanol, the silk proteins retain coiled coil structure and also develop beta-sheet structure; **D**. When drawn in methanol the proteins align and there is partial conversion of coiled coils to beta-sheet.

Our results suggest that the recombinant honeybee silk consists of coiled coils cross-linked by a network of β-sheets. The tensile strength of the material increases with increased β-sheet content in the fibers (Figure 4B and C and Table 1). A schematic showing the structural transformations of single honeybee recombinant proteins during the material fabrication process is shown in Figure 5. The levels of β-sheet and degree of alignment of the coiled coils can be controlled via the fabrication process. Preserving coiled coil structure by reducing the amount of draw results in a tough material with a reasonable strength. Whilst converting most coiled coil structure to β-sheet improves the strength of the material, it lowers the extensibility and thus results in a reduction in overall toughness.

### Role for the four proteins found in native silk

This study demonstrates that a single honeybee silk protein can adopt the native silk structure and achieve equivalent mechanical properties to material generated from the four paralogous honeybee silk proteins. It is therefore likely that the four paralogous coiled coil silk genes have been conserved for over 150 million years in social Hymenopteran lineages due to their protein products playing a critical role in the process of natural silk fabrication, rather than in conferring properties to the end product. In natural silk, production information encoded within the silk proteins drives the assembly of the proteins into higher order structures. For example, in honeybees the silk proteins self-assemble into coiled coils, which form filaments that congregate into fibrils, which then organise into tactoids forming a liquid crystal solution with the crystals aligned in the direction of flow through the spinneret [12].

At the protein sequence level it is apparent that there has been conservation of amino acids at many sites within each ortholog [4]. The greater stability upon concentration of coiled coils formed from all four paralogs over single proteins [13] implies that a proportion of these conserved residues dictate partner selection and protein orientation within the coiled coil, leading to preferential formation of a heterotetrameric structure. We propose that conserved residues on the surface of these heterotetrameric structures choreograph the organisation of the coiled coils into higher assemblies within the silk gland, most likely through ionic interactions or salt bridges. This macromolecular organisation into liquid crystals is essential as it generates a solution with sufficient overall viscosity to prevent capillary break-up of threads during extrusion of silk from the gland, yet with a reduced flow viscosity so that the animal is able to pass the solution through the spinneret.

### Summary

Honeybee silk proteins are amenable to recombinant production at full-length and high yield. Although natural honeybee silk is spun from four distinct proteins, in this study we demonstrate that silken fibres fabricated from a single recombinant silk protein can successfully reproduce the structural and functional properties of the native silk. The finding that the functional properties of the silk are not reliant on four proteins greatly simplifies the route to production for artificial honeybee silk.

## Materials and Methods

### Preparation of silk dope and fabrication of silk films and fibers

Proteins were expressed into the inclusion bodies of *E. coli* as described by Weisman et al. [13]. Inclusion bodies were purified using BugBuster Master Mix (Novagen) according to the manufacturer's protocol and stored in water at −20°C. Silk proteins were solubilised in 3% sodium dodecyl sulfate (SDS) with 2 h incubation at 60°C and stored at 4°C until required. Solutions of all four (AmelF1-4) honeybee silk proteins were obtained when required by mixing equimolar amounts of each protein. Most of the SDS was removed by adding KCl to a concentration of 300 mM, inducing precipitation of potassium dodecyl sulfate. This promoted refolding of the silk proteins and caused precipitation of nonrecombinant *E. coli* proteins from the solution. The precipitate was removed by centrifugation at 16 000 *g* for 5 minutes, leaving a clear solution of 1–3% silk protein at greater than 95% purity. The solution was dialysed against 10% PEG 8000 to concentrate the proteins and remove excess salt. Protein concentration was determined using the QuantiPro BCA assay kit (Sigma; St Louis, MO) and protein purity was checked by SDS-PAGE. SDS concentration was determined according to Rosconi *et al.* [36] and corrected for protein binding. Protein films were cast onto glass slides from concentrated protein solutions and dried in air.

Silken fibers were fabricated by extrusion of approximately 3% protein solutions through 10–15 cm of capillary tubing (65–100 µm diameter) at a rate of 4 m.min^−1^ into a 30 cm deep methanol/water bath containing between 50–80% methanol. This procedure resulted in continuous production of even, opaque threads. Coagulation did not occur at or below 40% methanol, and at methanol concentrations above 80% the protein coagulated on the tip of the capillary tube. Extruded threads were stored in the coagulation bath. In order to confirm the presence of all four proteins in fibres generated from solutions of four proteins, the mass of fibres generated from known amounts of solution containing all four proteins was determined and shown to be in agreement with the amount of protein in the solution used to fabricate the fibres. As there is no detectible loss of protein during fabrication, all four proteins must be present in the fibres at the same levels that they were found in the protein solution. For mechanical and structural analysis, the threads were cut to lengths of approximately 10 cm and dried in air by draping over the top of a beaker or Petri dish, where they formed translucent threads of 30–50 µm in diameter, with the diameter depending on both the diameter of the capillary tube used to generate the threads and the initial protein dope concentration. When required the dried threads were placed in a 90% methanol bath, drawn by hand to up to four times their original length, and air-dried again.

### Dynamic Light Scattering (DLS)

DLS measurements were performed on a Malvern Zetasizer (Malvern, UK) instrument at 25°C. The reported particle hydrodynamic diameters were measured from three samples using automatically optimized measurement conditions and protein mode analysis. In order to predict the hydrodynamic diameter of a honeybee silk coiled coil in solution, we looked at two limiting conditions. As the upper limit for the particle size of an AmelF3 coiled coil, if the proteins were entirely coiled coil, a single assembly could be approximated as rod-like since the persistence length of coiled coils is >100 nm, for example as described by Young et al. [37]. Assuming a 0.148 nm rise per residue for a coiled coil of 315 residues, a native-like tetrameric coiled coil of radius 1.2 nm, and a single hydration layer of thickness 0.4 nm, we calculate the length (L) and radius (r) of the rod and hence the expected hydrodynamic radius (R_H_) [38]:
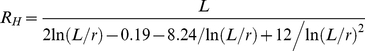
giving a hydrodynamic diameter of 18.2 nm. As the lower limit for the particle size for an AmelF3 coiled coil, if the “coiled coil” were entirely random coil we could calculate the expected hydrodynamic radius of a single unfolded protein containing 315 residues [39]:
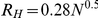
then for a tetramer increase volume by a factor of 4 which increases radius by ^3^√ 4, giving a hydrodynamic diameter of 15.8 nm. We therefore expect the hydrodynamic diameter of an AmelF3 tetramer that has a combination of coiled coil and random coil structure in solution to be approximately 16–18 nm.

### Circular dichroism

Circular dichroism spectra of honeybee AmelF3 solutions (0.12%) held in 0.01 mm path length sandwich quartz cell (Nova Biotech, El Cajon, CA) were collected using a AVIV Model 410 spectrophotometer (AVIV Biomedical, Inc., Lakewood, NJ) with a temperature controller. All samples were scanned at 25°C with a 1 nm bandwidth from 260 nm to 180 nm, and the results were averaged from four repeated experiments.

### Differential Scanning Calorimeter

Honeybee silk films (each about 5 mg) were encapsulated in vented aluminum pans and heated in a TA Instruments Q100 Differential Scanning Calorimeter (New Castle, DE), with purged dry nitrogen gas flow (50 mL.min^−1^), and equipped with a refrigerated cooling system. Differential Scanning Calorimetry measurements were performed at a heating rate of 2°C.min^−1^. Temperature-modulated Differential Scanning Calorimetry (TMDSC) measurements were also performed at a heating rate of 2°C.min^−1^ with a modulation period of 60 s and temperature amplitude of 0.318°C.

### Mechanical analysis of fibers

Undrawn or drawn fibers were mounted across a 2 mm gap on paper frames, fixed at either end with epoxy glue, and examined on an optical microscope to determine the gauge length and diameter of each fiber. Tensile measurements were carried out on an Instron Tensile Tester model 4501 at a rate of 2.5 mm.min^−1^. Tests were conducted in air at 21°C and 65% relative humidity. Fibres were examined for defects by light microscopy before mechanical testing. Data from fibres that broke during testing at the mounting points were excluded, all other data were included (7–9 fibres from each treatment, Table 1).

### Structural analysis of films and fibers

Infrared spectroscopy of the honeybee films was performed with a Jasco (Japan) FT/IR-6200 Spectrometer, equipped with a deuterated triglycine sulfate detector and a multiple reflection, horizontal MIRacle ATR attachment fitted with a Ge crystal (Pike Tech., Madison, WI). The instrument was continuously purged by nitrogen gas to eliminate the spectral contributions of atmospheric water vapor. For each measurement, 128 scans were co-added and Fourier transformed employing a Genzel-Happ apodization function to yield spectra with a nominal resolution of 4 cm^−1^. The wavenumber ranged from 600 to 4000 cm^−1^. To identify secondary structures of protein samples from the absorption spectra, we obtained the peak positions of the Amide I region (1595∼1705 cm-1) absorption from Fourier self-deconvolution performed using the Opus 5.0 software (Bruker) from Bruker Optics Corp. (Billerica MA), as described previously [22], [23].

Raman spectra were obtained using an inVia confocal microscope system (Renishaw, Gloucestershire, UK) with 514 nm excitation from a Stellar-Pro ML/150 argon ion laser (Modu-Laser, Centerville, Utah). Fibers mounted in a similar manner as for the mechanical strength analysis were analyzed through a x20 (0.40 n.a.) objective. An incident laser power of 6 mW was employed. Extended spectra of fibres were collected over the range 3200 to 100 cm^-1^ and averaged over at least 5 accumulations, each with an exposure time of 20 seconds. The Raman spectrum of SDS was obtained by supporting the sample on a glass microscope slide. Three accumulations, each with an exposure time of 10 seconds, were collected using a laser power of about 1 mW. The spectral resolution was ∼1 cm^−1^and the Raman shifts were calibrated using the 520 cm^−1^ line of a silicon wafer. Data collection was carried out using WiRE version 3.1 software. All post collection spectral processing was carried out using Grams AI software version 8.0.

## References

[1] Sutherland TD, Young J, Weisman S, Hayashi CY, Merrit D (2010). Insect silk: one name, many materials.. Annu Rev Entomol.

[2] Rudall KM, Florkin M, Mason HS (1962). Comparative Biochemistry Vol 4: Silk and other cocoon proteins.

[3] Atkins EDT (1967). A four-strand coiled-coil model for some insect fibrous proteins.. J Mol Biol.

[4] Sutherland TD, Weisman S, Trueman HE, Sriskantha A, Trueman JWH (2007). Conservation of essential design features in coiled coil silks.. Mol Biol Evol.

[5] Wilson EO (1971). The Insect Societies..

[6] Hepburn HR, Chandler HD, Davidoff MR (1979). Extensometric properties of insect fibroins: the green lacewing cross-β, honeybee α-helical and greater waxmoth parallel-β conformations.. Insect Biochem.

[7] Hepburn HR, Kurstjens SP (1988). The combs of honeybees as composite materials.. Apidologie.

[8] Zhang K, Duan H, Karihaloo BL, Wang J (2010). Hierarchical, multilayered cell walls reinforced by recycled silk cocoons enhance the structural integrity of honeybee combs. PNAS.

[9] Sutherland TD, Campbell PM, Weisman S, Trueman HE, Sriskantha A (2006). A highly divergent gene cluster in honey bees encodes a novel silk family.. Genome Res.

[10] Sezutzu H, Kajiwara H, Kojima K, Mita K, Tamura T (2007). Identification of four major hornet silk genes with a complex of alanine-rich and serine-rich sequences in *Vespa simillima xanthoptera* Cameron.. Biosci Biotechnol Biochem.

[11] Shi J, Lua S, Du N, Liu X, Song J (2008). Identification, recombinant production and structural characterization of four silk proteins from the Asiatic honeybee *Apis cerana*.. Biomaterials.

[12] Flower NE, Kenchington W (1967). Studies on insect fibrous proteins: the larval silk of *Apis*, *Bombus* and *Vespa* (Hymenoptera: Aculeata).. J Roy Micro Soc.

[13] Weisman S, Haritos VS, Church JS, Huson MG, Mudie ST (2010). Honeybee silk: Recombinant protein production, assembly and fiber spinning.. Biomaterials.

[14] Reynolds JA, Tanford C (1970). The gross conformation of protein-sodium dodecyl sulfate complexes.. J Biol Chem.

[15] Zhou NE, Kay CM, Hodges RS (1994). The net energetic contribution of interhelical electrostatic attractions to coiled-coil stability.. Protein Eng.

[16] Rabotyagova OS, Cebe P, Kaplan DL (2009). Self-assembly of genetically engineered spider silk block copolymers.. Biomacromolecules.

[17] Mattice WL, Riser JM, Clark DS (1976). Conformational properties of the complexes formed by proteins in sodium dodecyl sulfate.. Biochemistry.

[18] Parker W, Song P (1992). Protein structures in SDS micelle-protein complexes.. Biophys J.

[19] Meng F-G, Zeng X, Hong Y-K, Zhou H-M (2001). Dissociation and unfolding of GCN4 leucine zipper in the presence of sodium dodecyl sulfate.. Biochimie.

[20] Kameda T, Tamada Y (2009). Variable-temperature 13C solid-state NMR study of the molecular structure of honeybee wax and silk.. Int J Biol Macromol.

[21] Kameda T, Kojima K, Miyazawa M, Fujiwara S (2005). Film formation and structural characterization of silk of the hornet *Vespa simillima xanthoptera* Cameron.. *Z.* Naturforsch.

[22] Hu X, Kaplan D, Cebe P (2006). Determining beta-sheet crystallinity in fibrous proteins by thermal analysis and infrared spectroscopy.. Macromolecules.

[23] Hu X, Kaplan D, Cebe P (2008). Dynamic protein-water relationships during beta-sheet formation.. Macromolecules.

[24] Heimburg T, Schünemann J, Weber K, Geisler N (1999). FTIR-spectroscopy of multistranded coiled coil proteins.. Biochemistry.

[25] Peng XN, Chen X, Wu PY, Shao ZZ (2004). Investigation on the conformation transition of regenerated silk fibroin films under thermal treatment by two-dimensional (2D) correlation FT-IR spectroscopy.. Acta Chim Sin.

[26] Ishida M, Asakura T, Yokoi M, Saito H (1990). Solvent- and mechanical-treatment-induced conformational transition of silk fibroins studied by high-resolution solid-state ^13^C NMR spectroscopy.. Macromolecules.

[27] Tsukada M, Gotoh Y, Nagura M, Minoura N, Kasai N (1994). Structural changes of silk fibroin membranes induced by immersion in methanol aqueous solutions.. J Polym Sci Part B: Polym Phy*s*.

[28] Wilson D, Valluzzi R, Kaplan DL (2000). Conformational transitions in model silk peptides.. Biophys J.

[29] Kameda T, Kojima K, Togawa E, Suzutsu H, Zhang Q (2010). Drawing induced changes in morphology and mechanical properties of hornet silk gel films.. Biomacromolecules.

[30] Frushour BG, Koenig JK, Clark RSH, Hester RE (1975).

[31] Colthrup NB, Daly LH, Wiberly SE (1990). Introduction to Infrared and Raman Spectroscopy,.

[32] Feughelman M (1997). Mechanical Properties and Structure of Alpha-Keratin Fibres: Wool, Human Hair and Related Fibres,.

[33] Astbury WT, Woods HJ (1933). X-ray Studies of the Structure of Hair, Wool, and Related Fibers, II: Molecular Structure and Elastic Properties of Hair Keratin.. Phil Trans R Soc.

[34] Wortmann FJ, Zahn H (1994). The stress/strain curve of *a*-keratin fibres and the structure of the intermediate filament.. Text Res J.

[35] Kreplak L, Doucet J, Dumas P, Briki F (2004). New aspects of the alpha-helix to beta-sheet transition in stretched hard alpha-keratin fibers.. Biophysical J.

[36] Rusconi F, Valton E, Nguyen R, Dufourc E (2001). Quantification of sodium dodecyl sulfate in microliter-volume biochemical samples by visible light spectroscopy.. Anal Biochem.

[37] Li XC, Lehman W, Fischer S (2010). The relationship between curvature, flexibility and persistence length in the tropomyosin coiled-coil.. J Struct Biol.

[38] Young CY, Missel PJ, Mazer NA, Benedek GB, Carey MC (1978). Deduction of micellar shape from angular dissymmetry measurements of light scattered from aqueous sodium dodecyl sulfate solutions at high sodium chloride concentrations.. J Phys Chem.

[39] Damaschun G, Damaschun H, Gast K, Zirwer D (1998). Denatured states of yeast phosphoglycerate kinase.. Biochemistry (Moscow).

